# An Unusual Case of Bilateral Tibial Tubercle Avulsion With Complete Avulsion of the Unilateral Patellar Tendon

**DOI:** 10.7759/cureus.77319

**Published:** 2025-01-12

**Authors:** Ahmed Ghazi, John W Carlos, Kabir Sodhi, Mary Gething, Gurdip Chahal

**Affiliations:** 1 Trauma and Orthopaedics, Warwick Hospital, South Warwickshire University NHS Foundation Trust, Warwick, GBR; 2 Trauma and Orthopaedics, University of Warwick Faculty of Medicine, Warwick, GBR

**Keywords:** bilateral tibial tubercle injury, patellar tendon avulsion, simultaneous tibial tubercle and patellar tendon avulsion, skeletally immature tibial tubercle injuries, tibial tubercle avulsion

## Abstract

This is a case report of a 15-year-old male patient who presented with bilateral tibial tubercle avulsions and a unilateral complete patellar tendon stripping following a fall on both knees while playing football. The left knee was treated conservatively, while the right underwent open reduction internal fixation and patella tendon repair. The case was followed for one year postoperatively. Complete soft tissue and fracture healing were reported bilaterally. A modified Lysholm score of 84 compared to 95 pre-injury and a Tegner score of four compared to nine pre-injury were recorded. This case report highlights the absence of a consensus on the management of bilateral tibial tubercle avulsion fractures. To the best of our knowledge, it also marks the first documented instance of an additional concomitant unilateral patellar tendon avulsion. In this report, we have described the unique mechanism of injury and how a preoperative knee MRI scan is highly advisable to delineate associated injuries. We implemented a novel surgical technique combining internal fixation with a double-row-like technique addressing the bony and soft tissue elements, respectively. A supervised, gradually progressive rehabilitation program is recommended. This injury can massively affect an individual’s function level despite careful planning; therefore, we recommend future studies investigating potential underlying subclinical musculoskeletal disorders in similar cases that can help shape sport selection for different individuals.

## Introduction

Tibial tubercle avulsion fractures represent less than 1% of all physeal injuries in adolescents [1]. Patients presenting with concomitant patellar tendon rupture often report a sudden ‘pop’ at the point of injury, followed by immediate pain and knee effusion. This typically results in an inability to bear weight. However, the main indicators of patellar tendon rupture on examination include a palpable defect directly inferior to the patella, decreased range of motion, and, crucially, the loss of active knee extension or inability to maintain passive knee extension [2].

These fractures typically occur alongside the closure of the proximal tibial physis in adolescent males, as described by the Ehrenborg stages of tibial tubercle maturation [3]. This period of increased vulnerability is due to the excess force through the quadriceps tendon, which can cause the avulsion of the tibial tuberosity. The transition from fibrocartilage to columnar cartilage in the proximal tibia further indicates that this area is more susceptible to trauma. This is because the columnar cells of the epiphyseal physis are truncated and poorly developed. Additionally, the anatomical insertion of the quadriceps femoris into the proximal tibial tubercle predisposes it to injury, as the extensor mechanism of the knee is unparalleled by brittle columnar cartilage [4].

Tibial tubercle fractures typically occur while jumping or landing from a height. They can also occur during high-stress hyperflexion of the knee joint due to the increased power of both the quadriceps muscle and the extensor mechanism relative to bone maturity. Despite the lack of known predisposing factors, many cases in the literature indicate that Osgood-Schlatter’s disease is the most common potentiator [5].

Due to the rarity of tibial tubercle avulsion fractures, there is no standardised classification in the literature. However, three main classifications have been employed: the Watson-Jones classification, the Ogden classification, and the Salter-Harris classification. The Watson-Jones and Ogden classifications are specific to tibial tubercle avulsion fractures, with the Ogden classification further developing the criteria of the Watson-Jones classification. The Salter-Harris classification is widely used for fractures involving a single physis, unlike the tibial tubercle, although it is also periodically utilised to describe tibial tubercle avulsion fractures [6].

Simultaneous bilateral tibial tubercle avulsion fractures are particularly rare, with very few cases reported in the literature. Concomitant patellar tendon rupture in a tibial tubercle avulsion fracture is also exceptionally rare, with only 16 known previous cases [7]. The combination of simultaneous bilateral tibial tubercle avulsion and unilateral patellar tendon rupture has not been previously reported.

In the event of a concomitant patellar tendon rupture, surgical intervention is required. There is no standardised surgical technique for such a rare injury. Open reduction and internal fixation are typically preferred to treat the fracture, with good clinical outcomes and recovery scores consistently observed in patients. To treat the patellar tendon rupture, the Krakow method is used to secure the patellar tendon to the tibial tubercle, with a transosseous tunnel serving as a tether for the sutures [8].

Following treatment, post-surgical outcomes for tibial tubercle avulsion fractures are documented considering the patient’s previous knee function and activity levels. Two evaluative measurement systems are most commonly employed: the Lysholm Gillquist score and the Tegner Activity scale. Postoperative resumption of functional capacity is assessed via the Lysholm score, which comprises eight focused subscales: pain, support needs, instability, locking, stair climbing, squatting, swelling, and limp. The Tegner scale appraises activity level concerning work and sporting activities, using a score of 0-10, where 0 designates disability and 10 represents participation in national or international competitive sports. A 2009 survey evaluating subjects with accepted normal knee function proposed the average Lysholm score to be 94/100 and the average Tegner activity level to be 5.7/10. However, these two systems are not infallible, as the Tegner scale fails to account for the strength of forces placed through the lower limbs by not considering the intensity of each sport. Successful rehabilitation following tibial tubercle avulsion fracture has been generally reported in the literature, and, depending on the grade of the fracture, return to normal activity level varies between two to six months post-surgical intervention [9-11].

## Case presentation

A 15-year-old, skeletally immature, fit and well, White male patient with a healthy body mass index (21 kg/m^2^) was presented to the accident and emergency (A&E) department via ambulance in bilateral lower limb vacuum immobiliser splints. He was suffering from severe bilateral knee pain with moderate swelling and an inability to bear weight following direct trauma from falling on both knees while playing football. The injury was a simple, direct fall on both knees due to stumbling, without sprinting or being tackled, followed by immediate bilateral knee hyperflexion.

The patient was given adequate analgesia to control his pain, and ice was already applied to both knees. On examination, the patient was vitally stable. He had moderate swelling in both knees, which was more severe on the right side. He had severe tenderness on both tibial tuberosities with a minimal range of motion in both knees due to pain. Peripheral neurovascularity to both feet was intact, and no signs of compartment syndrome were detected. He couldn’t perform a straight leg raise; however, there was no hip tenderness or pain. Foot and ankle examination was normal.

**Figure 1:**
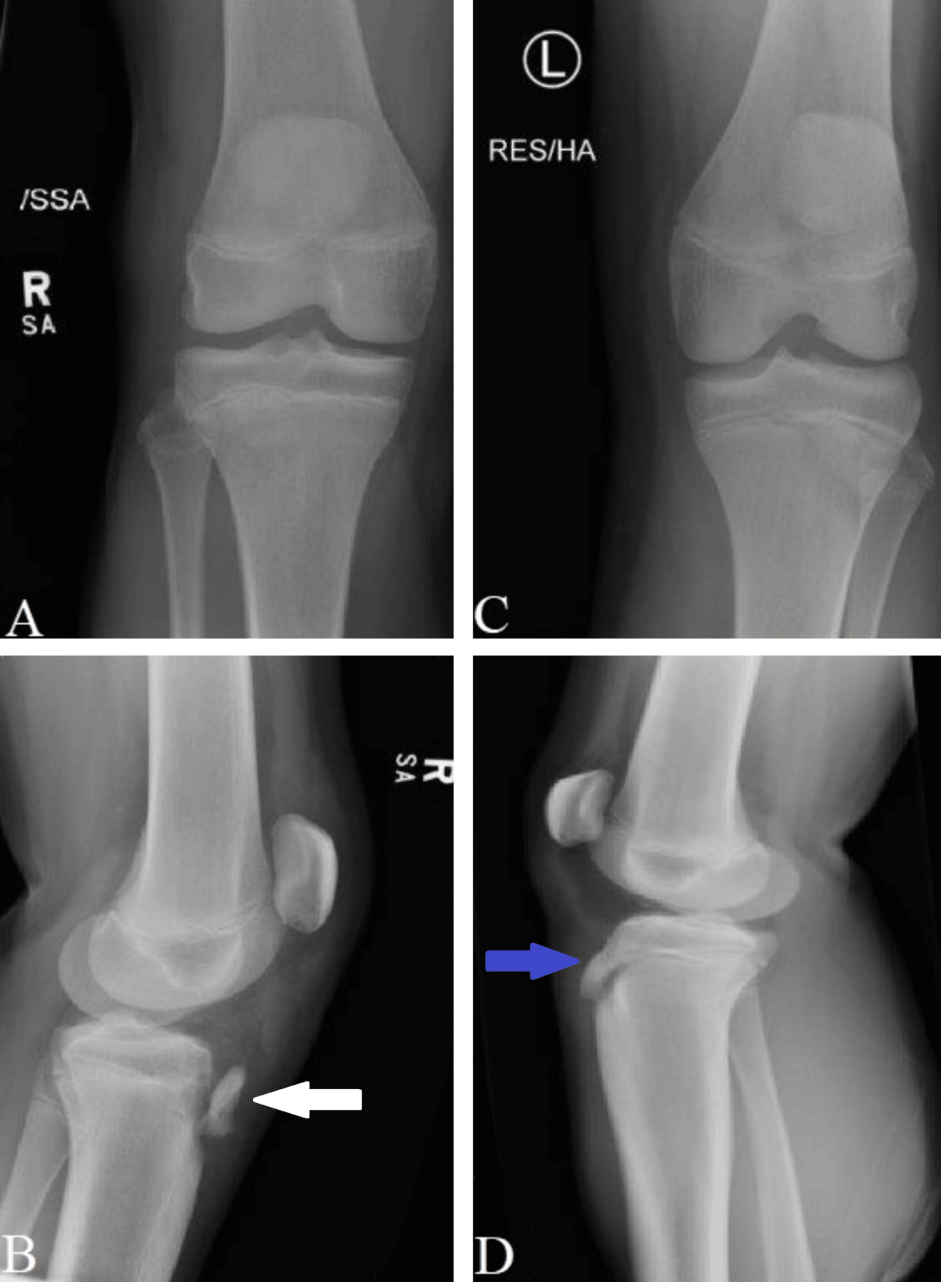
Preoperative X-rays Anteroposterior (AP) view of the right knee (A) and lateral view of the right knee (B) show a tibial tubercle flip avulsion fracture (white arrow). The AP view of the left knee (C) and lateral view of the left knee (D) show an avulsion fracture tibial tubercle minimally displaced (blue arrow).

The patient’s parents consented to use the injury footage and clinical pictures/scans for research and educational purposes while preserving the patient’s confidentiality. Bilateral knee and hip X-rays ruled out hip injuries and confirmed bilateral tibial tubercle avulsions (Figure 1).

The injury classification was type IA on the left side and type IIB on the right side, according to the Watson-Jones classification modified by Ogden et al. [1].

The patient was put in bilateral knee cricket splints and admitted to the paediatric orthopaedic ward. Strict elevation in Trendelenburg bed position and ice were applied in addition to guarding against compartment syndrome as it happens in 20% of cases, according to Yue et al., due to injury of the recurrent branch of the anterior tibial artery [2].

Due to the severity of the injury and the inability to assess both knees fully, the case was discussed via a multi-disciplinary team (MDT) with knee sports injury specialists. An urgent bilateral knee MRI scan was requested, which confirmed the integrity of the menisci, cruciates, collaterals, and osteochondral.

**Figure 2:**
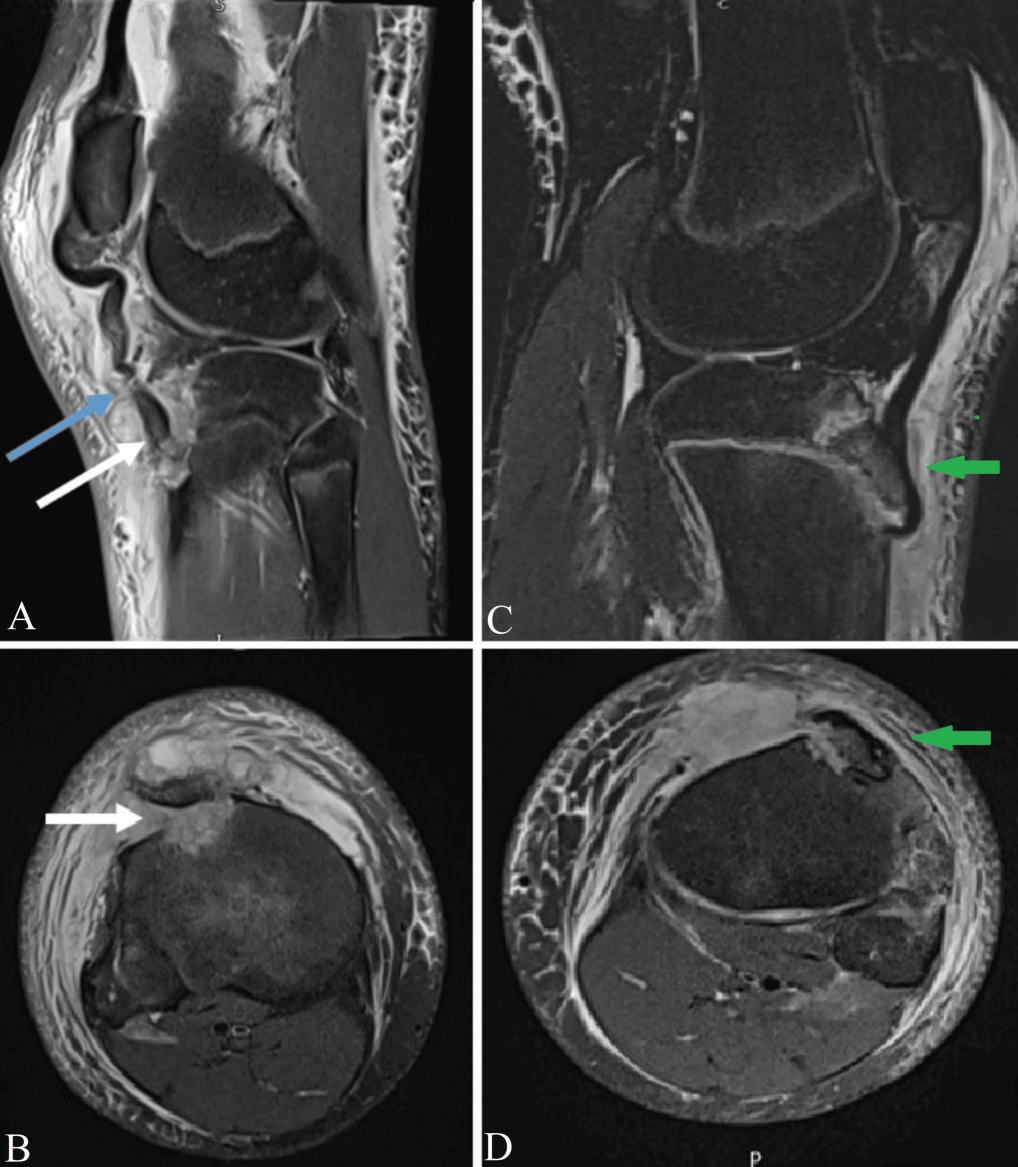
Preoperative MRI scan of both knees A: Sagittal view of the right knee shows double avulsion of the tibial tubercle flipped (white arrow) and the patellar tendon stripped of the tibial tubercle (blue arrow). B: Axial view of the right knee shows a tibial tubercle flipped avulsion fracture (white arrow). C: Sagittal view of the left knee shows a minimally displaced tibial tubercle avulsion fracture (green arrow). D: Axial view of the left knee shows a tibial tubercle minimally displaced avulsion fracture (green arrow).

However, the MRI scans (Figure 2) showed an isolated tibial tubercle avulsion fracture, minimally displaced on the left side. Astonishingly, it confirmed double avulsion on the right, where the tibial tubercle had an avulsion fracture, and the patellar tendon was completely stripped off its tibial tubercle insertion.

Reviewing the literature for such an injury on the right side, the double avulsion, confirmed no previous reporting nor classification.

**Figure 3:**
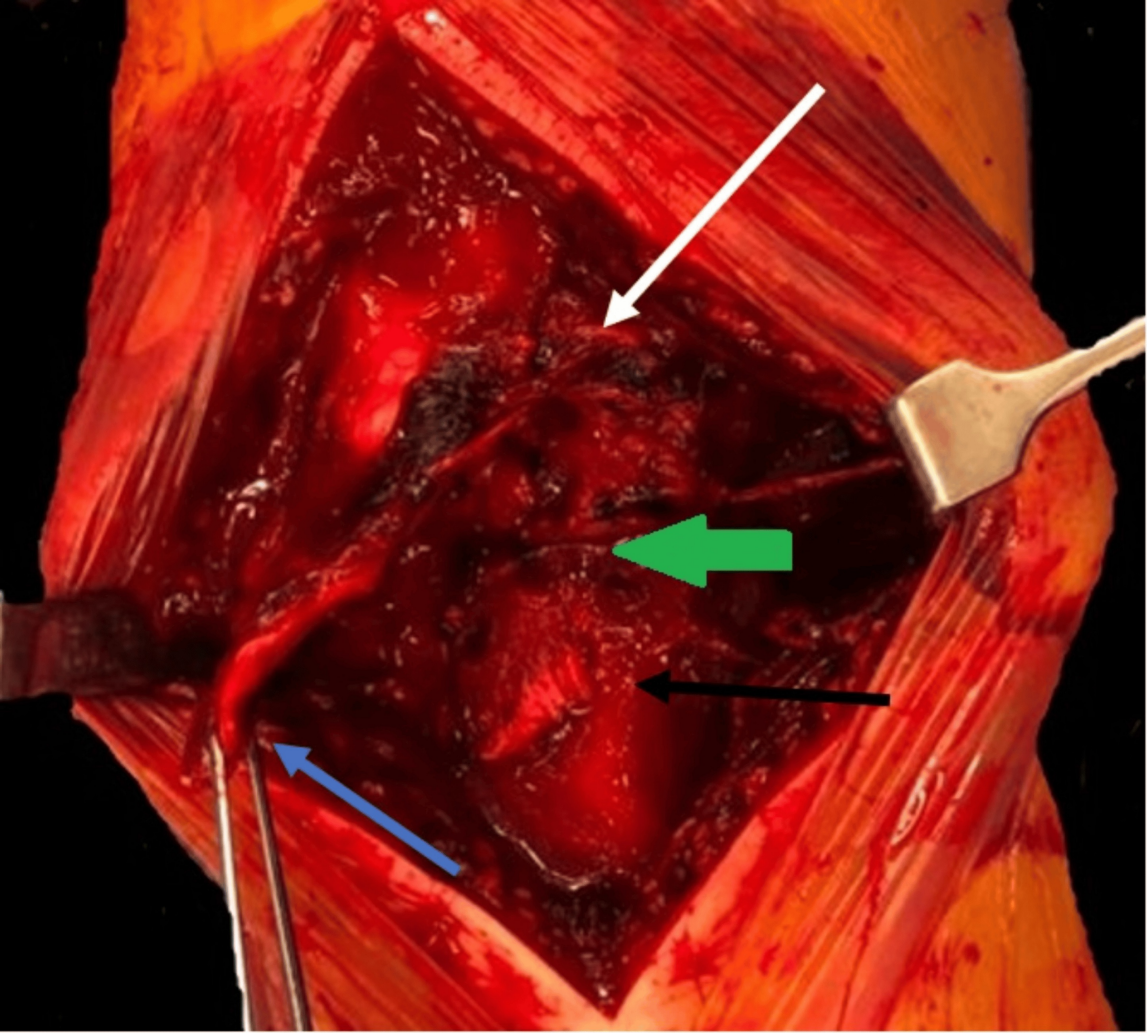
Intraoperative findings Patellar tendon strip avulsion (blue arrow); tibial tubercle avulsion fracture reduced (black arrow); tibial tubercle proximal pole still attached to the periosteum (green arrow); torn medial retinaculum (white arrow)

The MDT decided on conservative management of the left side while opting for surgical intervention on the right side. The patient underwent surgery on the right side, within 48 hours of admission, under general anesthesia. To prevent ischaemic revascularisation injury, which could lead to compartment syndrome, no tourniquet was used. During the procedure, a midline incision revealed that the patellar tendon was completely detached from the tibial tubercle, which was fractured and rotated 180 degrees. However, the periosteum remained attached to the proximal pole of the avulsed tibial tubercle fragment. Additionally, both the medial and lateral retinaculae were found to be torn (Figure 3).

**Figure 4:**
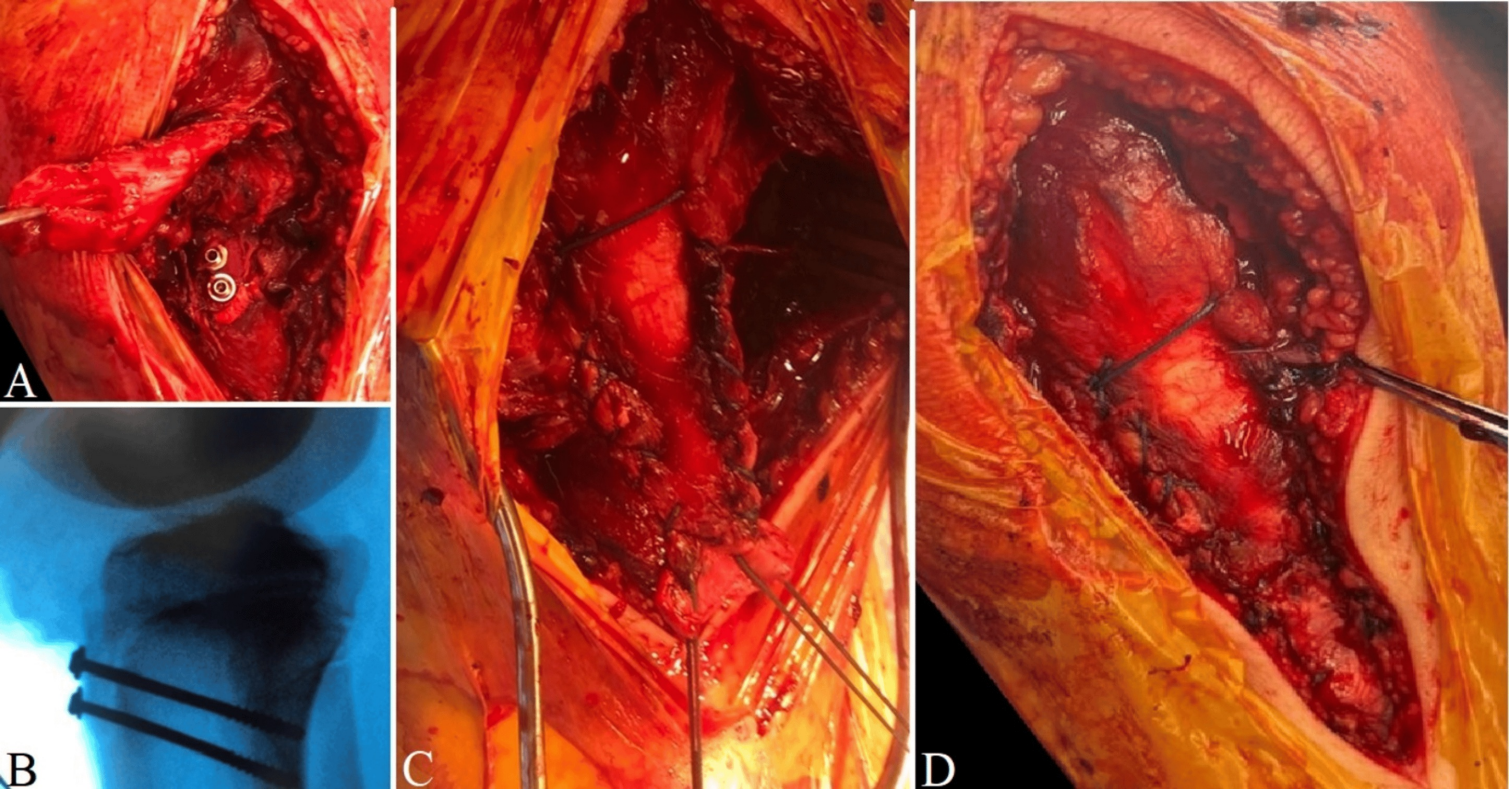
Intraoperative management A: Tibial tubercle open reduction internal fixation with two partially threaded 4 mm cannulated screws with small washers; B: Screw lengths and fracture reduction were confirmed under the image intensifier; C: The patellar tendon was repaired using a double whip stitch with Ethibond size 5 and two Depuy Mitek GII anchors; D: The medial retinaculum was repaired using a number 2 Orthocord.

The tibial tubercle was initially reduced, preserving the soft tissue attachment to its proximal pole. It was then fixed, under image intensifier guidance, using two partially threaded 4 mm cannulated screws with small washers inserted on guide wires. The avulsed patellar tendon was reattached to its footprint using the ‘double row’ technique, commonly employed in rotator cuff repairs, to create a broader fixation area. This involved using two size 5 Ethibond threads (DePuy Synthes, Raynham, MA, USA) in a double whip stitch technique, resulting in four limbs from the tip of the patellar tendon, with two on each corner. These were passed through two trans-osseous tunnels, one for each pair of Ethibond limbs, drilled to the desired tension to restore normal patellar height. Finally, two DePuy Mitek GII anchors (DePuy Synthes) were used to secure the patellar tendon to the proximal tibia, 1 cm proximal to the Ethibond trans-osseous tunnels, achieving an area of fixation similar to the double row technique. The retinaculae were then repaired using number 2 Orthocord (DePuy Synthes) (Figure 4), and the wound was closed in layers.

**Figure 5:**
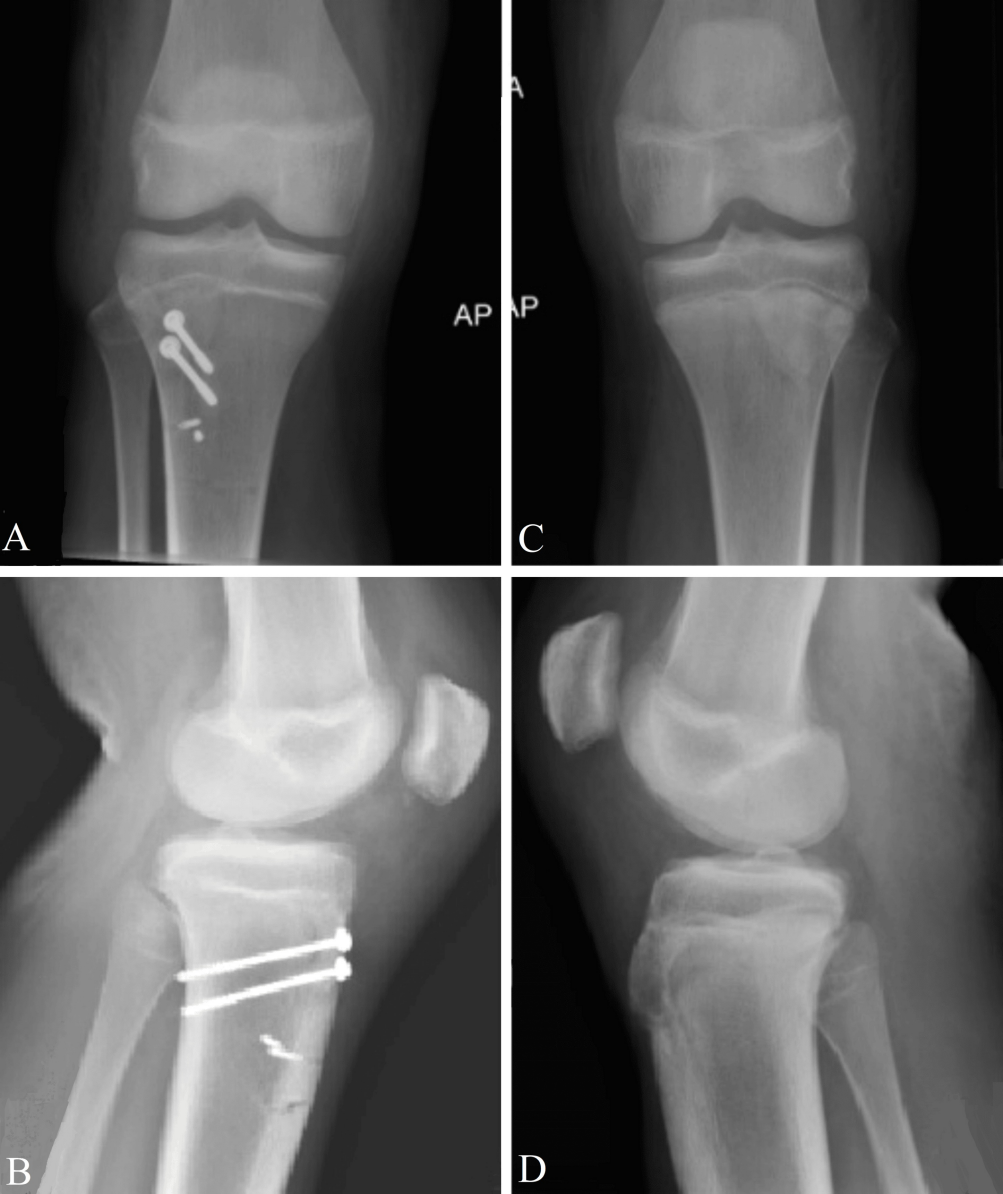
X-rays obtained six months postoperatively Anteroposterior (AP) view of the right knee (A) and lateral view of the right knee (B) show complete tibial tubercle fracture healing with screws, washers, and anchors in place. The AP view left knee (C) and lateral view of the left knee (D) show complete tibial tubercle fracture healing.

The postoperative rehabilitation plan was to allow full weight bearing with the knee locked in extension for four weeks, followed by a gradual increase in the range of motion by 30 degrees every two weeks. The patient was followed up at two weeks, six weeks, three months, six months (Figure 5), and one year postoperatively. The wound and fracture healed bilaterally without any acute or delayed development of compartment syndrome. However, the patient experienced minor numbness along the distribution of the superficial peroneal nerve on the right side two weeks postoperatively, which was attributed to a tight bandage. This issue was fully resolved by the six-week visit. The patient’s Tegner Score, which was nine pre-injury, dropped to 0 following the injury and surgery but gradually improved to three at six months and four at one year postoperatively. Similarly, the modified Lysholm score changed from 95 points preoperatively to 10 points post-injury/surgery, then improved to 59 points at 6 months and 84 points at one year postoperatively. These scores reflect the significant impact of such an injury on an individual’s functional level.

## Discussion

Epidemiology

To date, 30 cases have been reported worldwide with simultaneous bilateral tibial tubercle avulsion fractures, according to Giunchi et al. in their excellent narrative review and case reports [3]. This case is the first to be reported in the UK with bilateral simultaneous tibial tubercle avulsion and the first worldwide with additional unilateral double avulsion, where the patellar tendon was completely stripped off its insertion of the proximal tibia, and the tibial tubercle was avulsed separately. This emphasises the rarity of such a condition as already described in the introduction section, and therefore there’s no consensus on the management and rehabilitation plan. Therefore, we believe that this report will add to the pool of knowledge working towards building a management consensus.

Demographics

Although the number of reported cases is sparse, all the cases were male patients (96.7%) except one, according to Roy and Nag in their most extensive and detailed review of simultaneous bilateral tibial tubercle avulsion for the 60 years preceding 2013, with a mean age of 14 years. The current case report goes in line with that consensus [4], although there is no agreement on why it is more common in males. Some authors, like Giunchi et al., argue that it might be related to the anatomical variations related to the age of physeal closure, which is known to be a mean of 15 years in females and 17 in males. However, this cannot explain male predominance for such an injury; it would be expected to have variation in age of presentation between both sexes. Nevertheless, the same authors adopted the historical fact of increased male participation in sports, especially the more violent contact ones, to explain male predominance. This might explain the predominance in the past eras and would raise the argument of getting increased numbers of female cases to prove that theory in the coming years [3].

Mechanism of injury

In many of the reported cases, jumping is the main mechanism of injury, with the avulsion fracture happening during the landing phase due to sudden, forceful, eccentric quadriceps contraction that exerts tensile forces on the tibial tubercle physis beyond its ultimate strength limit, leading to the avulsion fracture as reported by Hanley et al. [5]. However, in the current case report, the mechanism of injury was direct trauma to both knees, as viewed on the injury footage, landing in a hyperflexed position. This showed a combination of shear and tensile forces simultaneously acting on the tibial tubercles bilaterally. This may explain the uniqueness of this case report injury pattern to produce a double avulsion on the right side, stripping off the patellar tendon of its tibial insertion together with tibial tubercle avulsion fracture and displacement.

Underlying comorbidities

There’s a current trend to think of an underlying aetiology that might subject some patients to such an injury as osteogenesis imperfecta, vitamin D deficiency, connective tissue disorders, and a pre-existing Osgood-Schlatter syndrome [6]. This was supported by Nicolini et al. in their case report and review of literature when they mentioned that these injuries are often associated with other orthopaedic disorders [7]. In contrast, in their 60-year review of literature, Roy and Nag reported that generally an underlying pathology is absent, but they referred to the presence of connective tissue disorder as a rare risk factor [4]. In the current case report, the patient was not investigated for any underlying pathology, relying on a clear past medical history. Similarly, the majority of the published reports didn’t focus on whether these cases need further thorough assessment to find out an underlying subclinical pathology or not.

Classification and prevalence

Tibial tubercle avulsion fracture was originally classified by Watson Jones in 1955, and this was modified by Ogden et al. in 1980 to further sub-classify each of the originally described three types into A-non-displaced & B-displaced +/- comminution [5, 8]. This has been extended further to include type IV in 1985 by Ryu and Debenham and type V in 2003 by McKoy and Stanitski [9]. Although the majority of the reported cases are type III (20 knees), the current case report is a type II on the right side, totalling nine knees and making type II the second most common, while it’s a type I on the left, totalling three knees, while six knees were reported as type IV [4].

Treatment and complications

Another current trend is to treat type I with a trial of closed reduction and conservative treatment, while other types should be treated with open reduction internal fixation [10]. The current case report goes in line with this trend. However, any type can be associated (1%-2%) with other knee injuries that cannot be found either on clinical assessment due to knee effusion and pain or on plain X-rays, which can underestimate the extent of soft tissue injuries [3]. Moreover, bilateral cases are associated with more complication rates compared to unilateral cases; this includes wound dehiscence, the need for hardware removal [11], knee rigidity, premature physeal closure, genu recurvatum, patellar malposition, non-union, malunion, bursitis, infection [12], and compartment syndrome requiring a low threshold for fasciotomy [13].

Implants and rehabilitation

To date, the choice of implants used for open reduction internal fixation is up to the surgeon’s preference; however, the most common types of implants used in the reported cases are either cannulated screws or tension band wiring [9]. In the current case report, different implants and techniques were used due to the uniqueness of the injury. In addition to the cannulated screws, a pull-out suture technique augmented with the double-row technique using anchors was chosen to treat the completely peeled patellar tendon insertion. This was the case as a direct repair of the tendon was not possible, and a more robust technique was needed to secure the repair.

Rehabilitation

Perhaps the most controversial part of managing similar cases is postoperative care. To date, there is no general agreement on whether to immobilise the knees in a cast or braces and no consensus on when to allow weight bearing and range of motion. In the current case report, the patient was allowed partial weight bearing with crutches in bilateral cricket splints, keeping the knees locked in full extension, allowing no range of motion for 4 weeks in total from surgery. This was followed by non-weight-bearing active flexion and passive extension in a hinged knee brace starting at 30 degrees for two weeks, then increasing by 30 degrees every two weeks until full range of motion while still maintaining the knee locked in full extension whilst walking. The difficulty lies mainly in the bilateral nature of the injury, as the patient cannot rely fully on one side to allow safe weight bearing; however, this approach can be adopted with fit and healthy young athletes who can comply with the protected weight bearing safely.

## Conclusions

This case report highlights the absence of a consensus on the management of bilateral tibial tubercle avulsion fractures. It also marks the first documented instance of an additional concomitant unilateral patellar tendon avulsion. In this report, we have described the unique mechanism of injury and how a preoperative knee MRI scan is highly advisable to delineate associated injuries. We implemented a novel surgical technique combining internal fixation with a double-row-like technique addressing the bony and soft tissue elements, respectively. A supervised, gradually progressive rehabilitation programme is recommended. This injury can massively affect an individual’s function level despite careful planning; therefore, we recommend future studies investigating potential underlying subclinical musculoskeletal disorders in similar cases that can help shape sport selection for different individuals.
